# Towards Further Understanding the Secondary Fracture during Spaghetti Bent Break

**DOI:** 10.3390/ma14010189

**Published:** 2021-01-02

**Authors:** Long Long, Yuxuan Zheng, Fenghua Zhou, Huilan Ren

**Affiliations:** 1 State Key Laboratory of Explosion Science and Technology, Beijing Institute of Technology, Beijing 100081, China; longlong@bit.edu.cn (L.L.); huilanren@bit.edu.cn (H.R.); 2 MOE Key Laboratory of Impact and Safety Engineering, Ningbo University, Ningbo 315211, China; zhengyuxuan@nbu.edu.cn

**Keywords:** flexural fracture, flexural stress wave, spaghetti, secondary fracture, bent break

## Abstract

When a brittle thin rod, such as a dry spaghetti stick, is bent beyond its flexural limit, it often breaks into more than two pieces, typically three or more. This phenomenon and puzzle has aroused widespread interest and discussion since its first proposal by Feynman. Previous work has partly explained the inevitability of the secondary fracture, but without any adjustable time parameter. In order to further understand this problem, especially the secondary fracture, in this paper we propose and study the dynamics of a half-infinite model to mimic the physics that a spaghetti stick is half-infinite under uniform bending. When the breaking process starts, a gradual release of initial moment of a linearly declining time at the free end, instead of a sudden release, is adopted, resulting in the introduction of a characteristic time parameter to the model and agrees better with the real situation. A specific analytical solution in terms of the excited bending moment using Euler–Bernoulli beam theory is derived, and that the gradual release of initial moment induces a burst of flexural waves, and these flexural waves locally increase the moment in the stick and progressively get to the maximum value, and then lead to the secondary fracture are concluded. The excited moment increases with time and distance, and has an asymptotic extremum value of 1.43 times initial moment. The gradual release in our model requires and gives certain distance and time when the excited bending moment reaches its extremum value, which provides a possibility to predict the detailed fracture parameters such as fragmentation length and time and thus to further understand the secondary fracture during spaghetti bent break.

## 1. Introduction

Multiple fracture of a brittle thin rod, such as a dry spaghetti stick, is a simple and intriguing puzzle in the field of fracture and failure mechanics that is originated from Miklowitz’s recognition and discussion in tensile fracture [1], followed by Phillips [2], Kolsky [3], and Kinra [4] in tension and Bodner [5], Kinra [6], and Schindler [7] in bending, and arouses widespread interest from Richard Feynman’s observation and discussion with Danny Hillis [8]. Feynman found that if a spaghetti stick is bent to break, it turns out that it will almost always break into three or more pieces instead of into halves. Feynman contemplated this process, allegedly ending up with a kitchen full of broken pasta, and left behind this famous Feynman puzzle.

To solve the puzzle, assume a full model problem to mimic the bent break process that a spaghetti stick is held at both ends and bent slowly. The stick breaks at time t=0 when the value of a certain physical parameter reaches its critical value, and the location of this first crack is that of the strongest defect. As Feynman mentioned and we observed from experiments, the breaking process will not stop at the first fracture event but continue to behave as a secondary fracture.

Nickalls [9,10] claimed to answer this puzzle in 1995 using tensor analysis, but the complexity was deconstructed in a letter published in 2005 by Audoly and Neukirch [11], for which they were later awarded an Ig Nobel prize.

**Figure 1:**
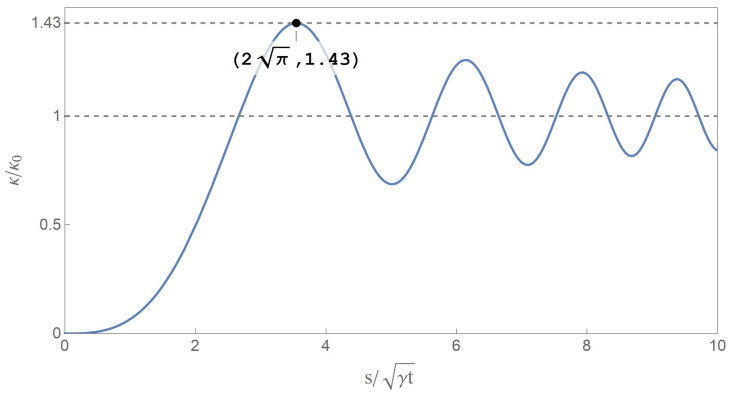
Self-similar solution in terms of curvature given by Audoly and Neukirch [11].

Audoly and Neukirch introduced a half-finite model problem of a stick of finite length *L* in which the release of the stick mimics the secondary fracture with the initial conditions that the stick is initially uniformly bent and at rest with the right end clamped and the left end free and applied a bending moment $M_{0}$, where $M_{0}$ plays a role of the internal moment transmitted across the section that is about to fail. Their boundary condition is that at time t=0, the left end is suddenly released as $M_{0}$ is removed instantaneously, and then yielded a self-similar solution in terms of curvature κ.

(1) $$\kappa \left(s,t\right)=2\kappa_{0}S\left(\frac{1}{\sqrt{2\pi }}\frac{s}{\sqrt{\gamma t}}\right)$$

where $\kappa_{0}$ represents the initial curvature, *s* length, *t* time, γ a material coefficient, and $S\left(y\right)=\int_{0}^{y}\sin\left(\frac{\pi z^{2}}{2}\right)dz$ the Fresnel sine integral function. The key property of this self-similar solution is that the excited curvature κ(s,t) will be significantly larger than the initial curvature $\kappa_{0}$, the extremum value of which is twice the maximum of the Fresnel sine integral (being 1.43). The sudden release of initial moment induces the flexural stress waves, increases the curvature locally and leads to a secondary fracture, as shown in Figure 1.

Audoly and Neukirch preliminarily explained Feynman’s puzzle and supported the inevitability of the multiple fracture when a spaghetti stick is bent to break. However, there still remains certain inaccuracies that require a deeper and further inspection and understanding to the secondary fracture event.

First, they assumed a model problem of a finite length *L* and misleading clamping conditions at s=L [11]: $\kappa_{,s^{2}}\left(L,t\right)=0$ and $\kappa_{,s^{3}}\left(L,t\right)=0$, where a comma in the indices denotes a partial derivative, but actually the clamping conditions should be $w_{,s^{2}}\left(L,t\right)=0$ and $w_{,s^{3}}\left(L,t\right)=0$, where *w* denotes displacement perpendicular to the neutral axis, and $\kappa =w_{,s^{2}}$ under the framework of Euler–Bernoulli (EB) beam theory. Next, they actually imposed infinite rather than finite boundary conditions to get to the self-similar solution of Equation (1), indicating that the schematic of their model shown in Figure 1 of [11] is misleading. Moreover, on the other hand, a self-similar solution implies an infinite boundary condition rather than a clamped boundary condition, which also implies that the length of the stick cannot be *L*. However, the self-similar solution given by Audoly is proper and reasonable, which is a special case in our model problem that will be discussed later in this paper, and is admissible enough to explain the inevitability of the secondary fracture.

Second, due to the limitation of the self-similar solution, Equation (1) lacks adjustable time parameter and accordingly the intrinsic fragmentation time and fragmentation length of the secondary fracture, two of which are of vital importance during the bent break process. Moreover, they assumed a boundary condition of sudden release of an initial moment to mimic the dynamics of the first fracture of the spaghetti bent break, which does not match the real situation that the transmission and completion of the crack of the first fracture process will not be completed instantaneously but require a certain time duration, and the certain time duration will controllingly influence the excitation and propagation of the flexural stress waves which will be discussed later.

**Figure 2:**
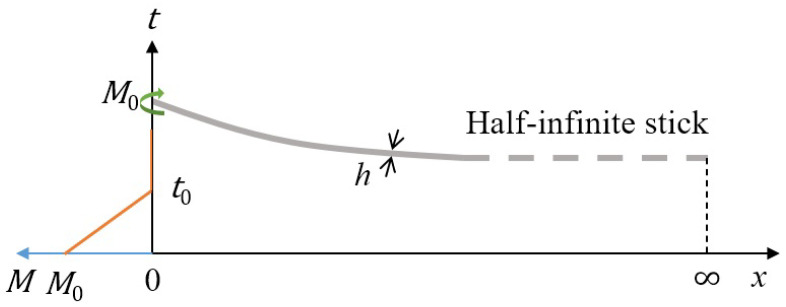
Schematic of the half-infinite gradual release model that mimics the secondary fracture.

In this paper, we assume a half-infinite model in which the release of a half-infinite spaghetti stick mimics the secondary fracture process and the initial condition is that the stick is initially uniformly bent and curved, and an initial bending moment $M_{0}$ is applied at the left end and the right end is infinite. The half-infinite model is schematized in Figure 2, where the thick gray line describes a half-infinite stick with a stick thickness of *h*. The ordinate always represents time, while the right direction of the abscissa axis represents distance *x*, from x=0 to infinity, and the left direction of the abscissa axis represents bending moment *M* at x=0, from its original value $M_{0}$ linearly declined to 0 within a release declining time $t_{0}$ in orange color.

We propose a model problem that a spaghetti stick is half-infinite and governed by EB beam theory, and adopt a gradual release boundary condition at the free end instead of a sudden release, which indicates the initial bending moment $M_{0}$ drops gradually to zero after a time duration $t_{0}$, as shown in Figure 2, thus bringing a characteristic time parameter $t_{0}$ related to adjustable fragmentation time and length to a non-self-similar solution, which improves the previous model, fixes the defect, is consistent with the real situation, and provides a positive assistance and guidance to the recently emerging micro- and nano-spaghetti mechanics [12].

## 2. Methodology

### 2.1. Control Equation

**Figure 3:**
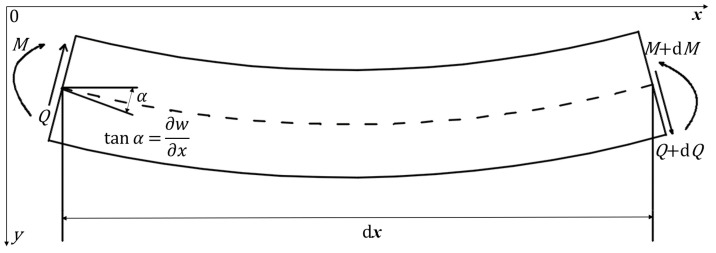
Schematic of basic equations using Euler–Bernoulli beam theory.

In this paper, we employ EB beam theory (Figure 3) as the governing control of our model, although we note that the EB theory does not account for certain shear effect described by complex beam theories such as Timoshenko beam theory. Indeed, the Timoshenko theory does provide a more accurate description of flexural stress waves with large wavenumber *k* compared with stick thickness *h*. However, Graff [13] remarks that in the regime of kh/4π<0.1, the distinction between the EB and Timoshenko beam theories can be negligible. In our model, the spaghetti stick is long thin with small thickness, and brittle with small wavenumber, so the EB beam theory is admissible.

Kinetic, kinematic, and material constitutive equations of EB beam theory shown in Figure 3 are as follows,

(2) $$\frac{\partial Q}{\partial x}=\rho A\frac{\partial v}{\partial t}$$

(3) $$\frac{\partial M}{\partial x}=Q$$

(4) $$v=\frac{\partial w}{\partial t}$$

(5) $$\omega =\frac{\partial^{2}w}{\partial x\partial t}$$

(6) $$\kappa =-\frac{\partial^{2}w}{\partial x^{2}}$$

(7) M=EIκ

where *M* represents bending moment, *x* axial displacement, *t* time, *Q* shear force on beam deformation, ρ mass density of beam, *A* cross-sectional area, *w* displacement perpendicular to neutral axis, *v*,ω linear speed and rotary speed, κ curvature, *E* elastic modulus, and *I* moment of inertia of the cross section.

Substituting kinematic Equations (4)–(6) and constitutive Equation (7) into kinetic Equations (2) and (3) gives the control equation:

(8) $$\frac{\partial^{2}M}{\partial t^{2}}+\frac{EI}{\rho A}\frac{\partial^{4}M}{\partial x^{4}}=0$$

Let $c_{0}=\sqrt{E/\rho }$ represent the elastic longitudinal stress wave velocity and $R=\sqrt{I/A}$ represent the radius of gyration of the cross section. For a rod with circular cross section of radius *r*, R=r/2, while for a rod with square cross section of side length *l*, $R=l/(2\sqrt{3})$. Thus, we can rewrite control equation Equation (8) to

(9) $$\frac{\partial^{2}M}{\partial t^{2}}+c_{0}^{2}R^{2}\frac{\partial^{4}M}{\partial x^{4}}=0$$

### 2.2. Initial and Boundary Conditions

In our model, we assume a half-infinite spaghetti stick from one end (x=0) and extended to another infinite end (x=∞) under uniformly bending at fixed curving rate. At the initial time t=0, the bending moment throughout the whole stick reaches to the critical value $M_{0}$, and then the breaking process starts and the bending moment at x=0 is about to drop linearly to zero with a release declining time duration $t_{0}$, as shown in Figure 2. Thus, we can give the initial and boundary conditions of the model. At t=0, the initial conditions are summarized as

(10) $$M\left(x,0\right)=M_{0}$$

(11) ω(x,0)=βx

where β is coefficient of rotary speed or curving rate, and Equation (11) means that the speed of rotation is proportional to the stick distance. The introduction of the curving rate β is one of the advantages and differences from previous assumption that the stick was initially uniformly bent and at rest, while we assume continuous curving at the curving rate β before the applied moment reaches $M_{0}$, while at t=0, the curving terminates and the initial moment unloads linearly as we will describe subsequently. β=0 reveals that the loading of the initial bending is quasi-static, while β>0 reveals that the loading of initial bending is dynamic or quenching which will be discussed in next paper.

Rewrite Equation (11) to the form of bending moment *M*:

(12) $$\frac{\partial M(x,0)}{\partial t}=-EI\frac{\partial \omega (x,0)}{\partial x}=-EI\beta$$

The boundary conditions are summarized as

(13) $$M\left(0,t\right)=\left\{\begin{array}{cc} M_{0}\left(1-t/t_{0}\right), & 0\leq t\leq t_{0}\\ 0, & t>t_{0} \end{array}\right.$$

(14) $$\frac{\partial M(0,t)}{\partial x}=0$$

(15) M(∞,t)=finitevalue

(16) $$\frac{\partial M(\infty ,t)}{\partial x}=0$$

where Equation (13) reveals gradually linearly declined release of initial moment $M_{0}$ with a time duration of $t_{0}$ at x=0, Equation (14) reveals no external applied shear force, and Equations (15) and (16) reveal the infinite boundary conditions that no external shear and a limited value of bending moment at the infinite end.

### 2.3. Nondimensionalization

In order to make our model and solution universal, and to simplify mathematical derivation, it is necessary to nondimensionalize our model problem, including the control equation and the initial and boundary conditions.

Define the characteristic parameters as follows, characteristic length *R*, characteristic time $R/c_{0}$, characteristic velocity $c_{0}$, characteristic stress *E*, characteristic moment $ER^{3}$, and characteristic rotary speed $c_{0}/R$. Then, the nondimensional parameters are obtained as original parameter divided by characteristic parameter:

(17) $$\bar{x}=\frac{x}{R},\bar{t}=\frac{tc_{0}}{R},\bar{M}=\frac{M}{ER^{3}},\bar{\omega }=\frac{\omega R}{c_{0}},\bar{A}=\frac{A}{R^{2}},\bar{I}=\frac{I}{R^{4}},\bar{M_{0}}=\frac{M_{0}}{ER^{3}},\bar{\beta }=\frac{\beta R^{2}}{c_{0}}$$

where a bar over a parameter means the nondimensionalized form of the corresponding parameter.

The nondimensional form of controlling equation is then

(18) $$\frac{\partial^{2}\bar{M}}{\partial {\bar{t}}^{2}}+\frac{\partial^{4}\bar{M}}{\partial {\bar{x}}^{4}}=0$$

The nondimensional form of initial conditions are

(19) $$\bar{M}\left(\bar{x},0\right)=\bar{M_{0}}$$

(20) $$\frac{\partial \bar{M}\left(\bar{x},0\right)}{\partial \bar{t}}=-\bar{A}\bar{\beta }$$

The nondimensional form of boundary conditions are

(21) $$\bar{M}\left(0,\bar{t}\right)=\left\{\begin{array}{cc} \bar{M_{0}}\left(1-\bar{t}/\bar{t_{0}}\right), & 0\leq \bar{t}\leq \bar{t_{0}}\\ 0, & \bar{t}>\bar{t_{0}} \end{array}\right.$$

(22) $$\frac{\partial \bar{M}\left(0,\bar{t}\right)}{\partial \bar{x}}=0$$

(23) $$\bar{M}\left(\infty ,\bar{t}\right)=\;\mathrm{finite}\;\mathrm{value}\;$$

(24) $$\frac{\partial \bar{M}\left(\infty ,\bar{t}\right)}{\partial \bar{x}}=0$$

Note that the boundary condition Equation (21) at x=0 is a linear piecewise function of time, suggesting that we can use the linear superposition principle to make our derivation simple and convenient. Define $\bar{M_{1}}\left(\bar{x},\bar{t}\right)$ as part of the excited bending moment $\bar{M}\left(\bar{x},\bar{t}\right)$ with an infinite declining boundary condition on the entire time axis even when $\bar{t}>\bar{t_{0}}$,

(25) $$\bar{M_{1}}\left(0,\bar{t}\right)=\bar{M_{0}}\left(1-\bar{t}/\bar{t_{0}}\right)$$

Thus, the excited bending moment in our model with a linear piecewise boundary condition can be written as

(26) $$\bar{M}\left(\bar{x},\bar{t}\right)=\bar{M_{1}}\left(\bar{x},\bar{t}\right)-H\left(\bar{t}-\bar{t_{0}}\right)\left[\bar{M_{1}}\left(\bar{x},\bar{t}-\bar{t_{0}}\right)-\bar{M_{0}}\right]$$

where H(t) is unit step function, $H\left(t\right)=\left\{\begin{array}{c} 1,t\geq 0\\ 0,t<0 \end{array}\right.$.

### 2.4. Solution to Our Model

Laplacian transform method is adopted to solve the nondimensionalized model problem. We first derive image function of excited bending moment using Laplacian transform in the frequency domain, and then derive primitive function using inverse Laplacian transform back in the time domain.

(27) $$\hat{M}\left(\bar{x},s\right)=L\left(\bar{M}\left(\bar{x},\bar{t}\right)\right)=\int_{0}^{+\infty }\bar{M}\left(\bar{x},\bar{t}\right)\exp\left(-s\bar{t}\right)d\bar{t}$$

(28) $$\bar{M}\left(\bar{x},\bar{t}\right)=L^{-1}\left(\hat{M}\left(\bar{x},s\right)\right)=\frac{1}{2i\pi }\int_{\alpha -i\infty }^{\alpha +i\infty }\hat{M}\left(\bar{x},s\right)\exp\left(s\bar{t}\right)ds$$

where $\hat{M}\left(\bar{x},s\right)$ denotes the image function in the frequency domain with frequency variable of *s*, and L,$L^{-1}$ are symbols of Laplacian transform and inverse Laplacian transform, respectively.

Laplacian transform of controlling equation Equation (18) is

(29) $$\frac{d^{4}\hat{M}\left(\bar{x},s\right)}{d{\bar{x}}^{4}}+s^{2}\hat{M}\left(\bar{x},s\right)-s\bar{M}\left(\bar{x},0\right)-\frac{d\bar{M}\left(\bar{x},0\right)}{d\bar{t}}=0$$

Using initial conditions Equations (19) and (20), we can get

(30) $$\frac{d^{4}\hat{M}\left(\bar{x},s\right)}{d{\bar{x}}^{4}}+s^{2}\hat{M}\left(\bar{x},s\right)-s\bar{M_{0}}-\bar{A}\bar{\beta }=0$$

General solution of Equation (30) usually contains four undetermined coefficients, but considering the infinite boundary condition Equations (23) and (24) that bending moment is finite-valued and no external shear force is applied, the four undetermined coefficients are simplified to two, and the simplified general solution of Equation (30) is then

(31) $$\hat{M}\left(\bar{x},s\right)=\frac{\bar{A}\bar{\beta }}{s^{2}}+\frac{\bar{M_{0}}}{s}+\exp\left(-\sqrt{\frac{s}{2}}\bar{x}\right)\left[c_{1}\sin\left(\sqrt{\frac{s}{2}}\bar{x}\right)+c_{2}\cos\left(\sqrt{\frac{s}{2}}\bar{x}\right)\right]$$

where $c_{1}$ and $c_{2}$ are undetermined coefficients, and they can be derived from the remaining boundary conditions Equations (22) and (25) of $\bar{M_{1}}$.

(32) $$c_{1}=c_{2}=-\frac{\bar{M_{0}}+\bar{A}\bar{\beta }\bar{t_{0}}}{s^{2}\bar{t_{0}}}$$

The final form of image function of $\hat{M_{1}}$ is

(33) $$\hat{M_{1}}\left(\bar{x},s\right)=\frac{\bar{A}\bar{\beta }}{s^{2}}+\frac{\bar{M_{0}}}{s}-\frac{\bar{M_{0}}+\bar{A}\bar{\beta }\bar{t_{0}}}{s^{2}\bar{t_{0}}}\exp\left(-\sqrt{\frac{s}{2}}\bar{x}\right)\left[\sin\left(\sqrt{\frac{s}{2}}\bar{x}\right)+\cos\left(\sqrt{\frac{s}{2}}\bar{x}\right)\right]$$

Now, we get the image function $\hat{M_{1}}\left(\bar{x},s\right)$ in the frequency domain, and then we are going to start Laplacian transform inversion to get the primitive function $\bar{M_{1}}\left(\bar{x},\bar{t}\right)$ back in the time domain. Note that the first two terms of Equation (33) can be easily inverse Laplacian transformed:

(34) $$L^{-1}\left(\frac{\bar{A}\bar{\beta }}{s^{2}}+\frac{\bar{M_{0}}}{s}\right)=\bar{A}\bar{\beta }\bar{t}+\bar{M_{0}}$$

Meanwhile, the last term of the image function Equation (33) is the product of two parts, suggesting that we can employ the method of the convolution law of Laplacian transform to get the primitive function. Given the known convolution law,

(35) $$L^{-1}\left[F\left(s\right)G\left(s\right)\right]=f\left(t\right)*g\left(t\right)=\int_{0}^{t}f\left(t-\tau \right)g\left(\tau \right)d\tau$$

where ∗ is the symbol of the convolution operation and F(s),G(s) represent two image functions in the frequency domain, while f(t),g(t) represent the corresponding primitive functions in the time domain.

We can find and get the following relations of Laplacian transform inversion from textbooks or manuals, such as [14]

(36) $$L^{-1}\left[\frac{\bar{M_{0}}+\bar{A}\bar{\beta }\bar{t_{0}}}{s^{2}\bar{t_{0}}}\right]=\frac{\bar{t}}{\bar{t_{0}}}\left(\bar{M_{0}}+\bar{A}\bar{\beta }\bar{t_{0}}\right)$$

(37) $$L^{-1}\left[\exp\left(-\sqrt{\frac{s}{2}}\bar{x}\right)\left\{\sin\left(\sqrt{\frac{s}{2}}\bar{x}\right)+\cos\left(\sqrt{\frac{s}{2}}\bar{x}\right)\right\}\right]=\frac{\bar{x}}{\sqrt{2\pi }}{\bar{t}}^{-\frac{3}{2}}\sin\left(\frac{{\bar{x}}^{2}}{4\bar{t}}\right)$$

According to convolution law of Laplacian transform, we have

(38) $$\begin{array}{cc} \; & L^{-1}\left[\left(\frac{\bar{M_{0}}+\bar{A}\bar{\beta }\bar{t_{0}}}{s^{2}\bar{t_{0}}}\right)\exp\left(-\sqrt{\frac{s}{2}}\bar{x}\right)\left\{\sin\left(\sqrt{\frac{s}{2}}\bar{x}\right)+\cos\left(\sqrt{\frac{s}{2}}\bar{x}\right)\right\}\right]\\ =\; & L^{-1}\left[\frac{\bar{M_{0}}+\bar{A}\bar{\beta }\bar{t_{0}}}{s^{2}\bar{t_{0}}}\right]*L^{-1}\left[\exp\left(-\sqrt{\frac{s}{2}}\bar{x}\right)\left\{\sin\left(\sqrt{\frac{s}{2}}\bar{x}\right)+\cos\left(\sqrt{\frac{s}{2}}\bar{x}\right)\right\}\right]\\ =\; & \left[\frac{\bar{t}}{\bar{t_{0}}}\left(\bar{M_{0}}+\bar{A}\bar{\beta }\bar{t_{0}}\right)\right]*\left[\frac{\bar{x}}{\sqrt{2\pi }}{\bar{t}}^{-\frac{3}{2}}\sin\left(\frac{{\bar{x}}^{2}}{4\bar{t}}\right)\right]\\ =\; & \int_{0}^{\bar{t}}\frac{\bar{t}-\tau }{\bar{t_{0}}}\left(\bar{M_{0}}+\bar{A}\bar{\beta }\bar{t_{0}}\right)\frac{\bar{x}}{\sqrt{2\pi }}\tau^{-\frac{3}{2}}\sin\left(\frac{{\bar{x}}^{2}}{4\tau }\right)d\tau \\ =\; & \left(\bar{A}\bar{\beta }+\frac{\bar{M_{0}}}{\bar{t_{0}}}\right)\left[\bar{t}-2\bar{t}S\left(\frac{\bar{x}}{\sqrt{2\pi \bar{t}}}\right)-\frac{{\bar{x}}^{2}}{2}+{\bar{x}}^{2}C\left(\frac{\bar{x}}{\sqrt{2\pi \bar{t}}}\right)-\bar{x}\sqrt{\frac{2\bar{t}}{\pi }}\sin\left(\frac{{\bar{x}}^{2}}{4\bar{t}}\right)\right] \end{array}$$

where $S\left(y\right)=\int_{0}^{y}\sin\left(\frac{\pi z^{2}}{2}\right)dz$ and $C\left(y\right)=\int_{0}^{y}\cos\left(\frac{\pi z^{2}}{2}\right)dz$ are the Fresnel sine integral function and Fresnel cosine integral function, respectively. Then, we get the primitive function solution of $\bar{M_{1}}$,

(39) $$\begin{array}{c} \bar{M_{1}}\left(\bar{x},\bar{t}\right)=\bar{M_{0}}+\bar{A}\bar{\beta }\bar{t}\\ -\left(\bar{A}\bar{\beta }+\frac{{\bar{M}}_{0}}{\bar{t_{0}}}\right)\left[\bar{t}-2\bar{t}S\left(\frac{\bar{x}}{\sqrt{2\pi \bar{t}}}\right)-\frac{{\bar{x}}^{2}}{2}+{\bar{x}}^{2}C\left(\frac{\bar{x}}{\sqrt{2\pi \bar{t}}}\right)-\bar{x}\sqrt{\frac{2\bar{t}}{\pi }}\sin\left(\frac{{\bar{x}}^{2}}{4\bar{t}}\right)\right] \end{array}$$

We can finally get the primitive function solution with the linear piecewise boundary condition to our model using linear superposition principle as shown in Equation (26), and we write it again for reading convenience.

(40) $$\bar{M}\left(\bar{x},\bar{t}\right)=\bar{M_{1}}\left(\bar{x},\bar{t}\right)-H\left(\bar{t}-\bar{t_{0}}\right)\left[\bar{M_{1}}\left(\bar{x},\bar{t}-\bar{t_{0}}\right)-\bar{M_{0}}\right]$$

Note that, $\bar{\beta }$ represents curving rate indicating the intensity of the initial bending or the initial bending speed, and greater value of $\bar{\beta }$ reveals intensely dynamic bending, but in our model the bent of the half-infinite spaghetti stick is assumed to be quasi-static, implying $\bar{\beta }=0$. Therefore, we can rewrite $\bar{M_{1}}$ to a simpler form hereafter, and related issues of dynamic bending will be discussed in next paper.

(41) $$\bar{M_{1}}\left(\bar{x},\bar{t}\right)=\bar{M_{0}}-\frac{{\bar{M}}_{0}}{\bar{t_{0}}}\left[\bar{t}-2\bar{t}S\left(\frac{\bar{x}}{\sqrt{2\pi \bar{t}}}\right)-\frac{{\bar{x}}^{2}}{2}+{\bar{x}}^{2}C\left(\frac{\bar{x}}{\sqrt{2\pi \bar{t}}}\right)-\bar{x}\sqrt{\frac{2\bar{t}}{\pi }}\sin\left(\frac{{\bar{x}}^{2}}{4\bar{t}}\right)\right]$$

## 3. Results and Discussions

### 3.1. Quantitative Analysis

Now, we are going to investigate the influence of the introduction of the gradual release declining time $\bar{t_{0}}$ on the induced flexural stress waves and excited bending moment and thus the influence on the process of the secondary fracture during spaghetti bent break.

The physical meaning of $\bar{t_{0}}$ is the time duration of the gradually declined release of initial bending moment at the free end dropping from original value ${\bar{M}}_{0}$ to zero. The greater value of $\bar{t_{0}}$ reveals a longer time duration in the boundary condition at the free end, and vice versa.

First, let us consider one of the special cases. We can see from Equations (40) and (41) that when the value of $\bar{t_{0}}$ approaches to zero, or to say, when the gradually declined release boundary condition of our model is simplified to a special case—the sudden release boundary condition case, the solution is accordingly simplified to

(42) $$\bar{M}\left(\bar{x},\bar{t}\right)=2\bar{M_{0}}S\left(\frac{\bar{x}}{\sqrt{2\pi \bar{t}}}\right)$$

Equation (42) is the so-called self-similar solution in terms of excited bending moment with variables of distance and time, the form of which is the same as Equation (1). It can be easily figured out that the extremum value of the excited bending moment from Equation (42) is twice the maximum of the Fresnel sine integral although lacking of any adjustable time parameter, that is, the excited bending moment will always get to 1.43 times initial moment somewhere in the half-infinite stick regardless of how short the propagate time will be, and then lead to a secondary fracture. Thus, the self-similar solution can only demonstrate the inevitability of a secondary fracture, but intrinsically indicates an infinite stress wave speed, which is not in line with the real situation.

Second, let us assume some typical non-zero values of $\bar{t_{0}}$ to investigate the difference and improvement of the introduction of $\bar{t_{0}}$ and whether varying values of $\bar{t_{0}}$ affect the excited bending stress waves and their propagation and how.

**Figure 4:**
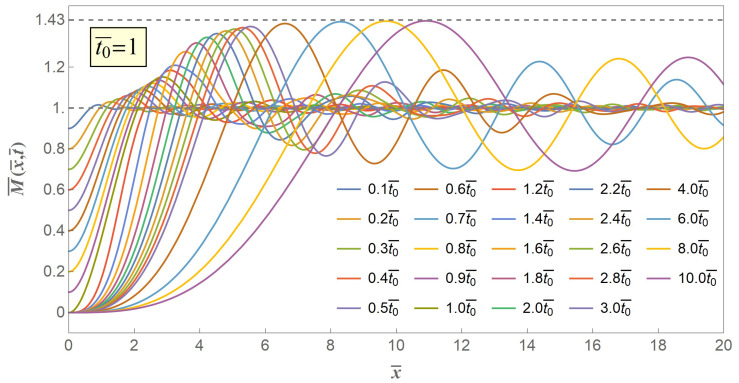
Schematic of excited bending moment versus distance when $\bar{t_{0}}=1$.

Assuming $\bar{t_{0}}=1$, we can get excited bending moment versus distance curves in progressive time coordinates as shown in Figure 4. The gradually declined release of initial bending moment excites a series of flexural stress waves from the free end of the stick, and these flexural stress waves locally increase the excited bending moment to beyond a critical limit and eventually lead to a secondary fracture. The excited stress wave propagates through the stick, but will not affect the physical state far away from the wave front. The excited bending moment at the free end of the stick gradually declines with the propagation time from initial value down to zero when $0\leq \bar{t}\leq \bar{t_{0}}$, which is consistent with the boundary condition Equation (14), and propagates through the whole stick without any external value at the free end when $\bar{t}>\bar{t_{0}}$. Under any time coordinate, the bending moment excited from the gradual release of the initial moment always monotonously grows to a maximum value, and this maximum value increases with time, but will not increase indefinitely. It can be seen from Figure 4, and also can be proved mathematically from the analytical solution, that the maximum excited bending moment ${\bar{M}}_{\max}$ has an asymptotic limit value of twice the maximum of the Fresnel sine integral, being 1.43. The difference between non-zero valued $\bar{t_{0}}$ case and approaching zero valued $\bar{t_{0}}$ case lies on the necessity of a certain propagate time when the excited moment grows to some certain value. Therefore, the introduction of gradually release boundary condition of our model brings an adjustable characteristic time parameter, and accordingly provides a possibility to predict the intrinsic fragmentation time and fragmentation length.

**Figure 5:**
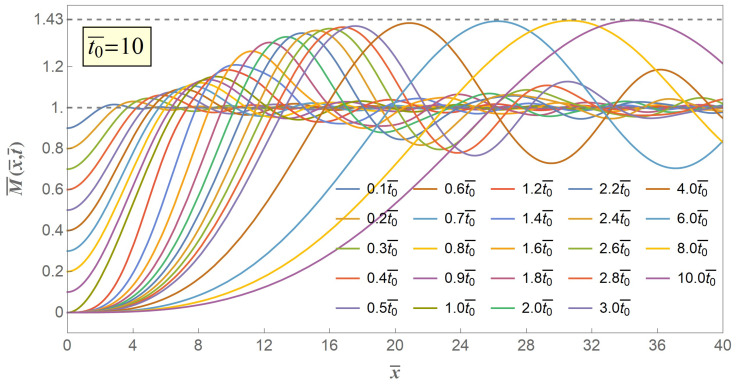
Schematic of excited bending moment versus distance when $\bar{t_{0}}=10$.

**Figure 6:**
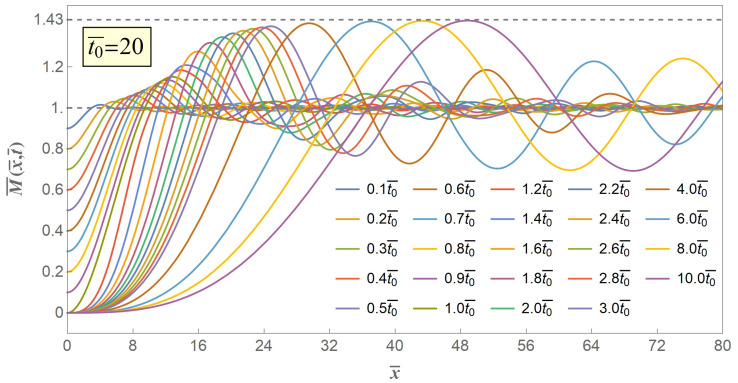
Schematic of excited bending moment versus distance when $\bar{t_{0}}=20$.

Then, we extend the value of $\bar{t_{0}}$ from 1 to greater numbers, and cases of two typical values of release declining time $\bar{t_{0}}=10$ and $\bar{t_{0}}=20$ are illustrated in Figure 5 and Figure 6, respectively.

As can be seen from Figure 5 and Figure 6, the linearly gradually declined release with greater-valued time duration likewise excites a series of flexural stress waves propagating from the free end of the stick to the infinite end and these flexural stress waves locally increase the excited bending moment to beyond a critical limit and eventually lead to a secondary fracture. Under any time coordinate, the excited bending moment always monotonously grows to a maximum value, and the maximum value increases with propagation time to an asymptotic extremum value of ${\bar{M}}_{\max}$.

Figure 4, Figure 5 and Figure 6 show the excited flexural waveform curves at progressive time coordinates, and we can figure out that at the very beginning of both distance and time the excited bending moment will rise to beyond its initial value, that is, at the very beginning as long as the flexural stress wave is excited, the secondary fracture is about to happen, provided that once the bending moment exceeds it initial value ${\bar{M}}_{0}$ a secondary fracture will start up. However, experience and experiments from us and other scholars [11,15] suggest that the secondary fracture will take place at a point with some certain distance from the first fracture point, which reminds that the crack criterion of the secondary fracture should be not the same as the quasi-static first fracture.

**Figure 7:**
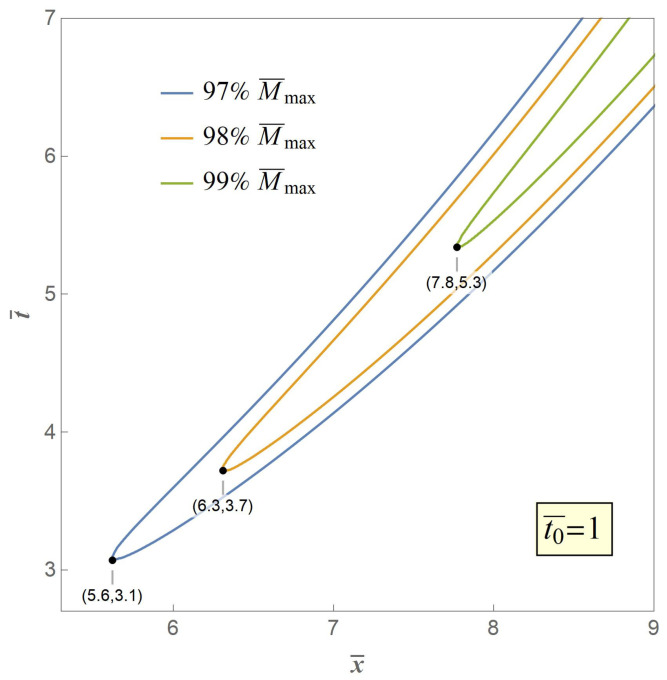
Contour curves when $\bar{M}=97\%,98\%,99\%{\bar{M}}_{\max}$ at $\bar{t_{0}}=1$.

**Figure 8:**
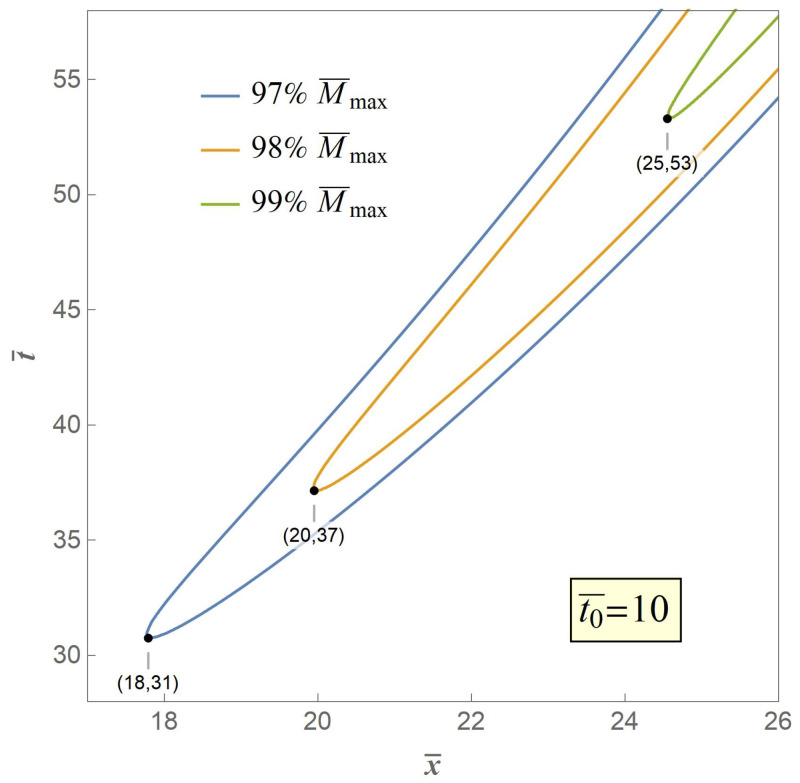
Contour curves when $\bar{M}=97\%,98\%,99\%{\bar{M}}_{\max}$ at $\bar{t_{0}}=10$.

**Figure 9:**
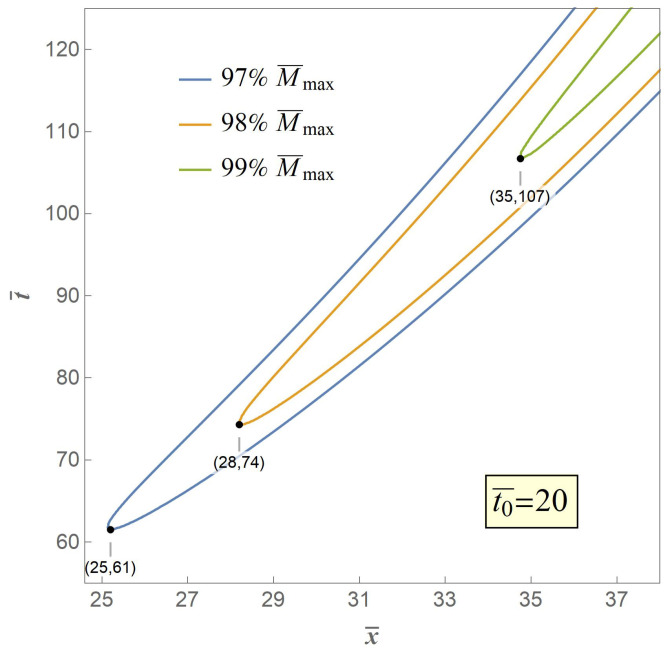
Contour curves when $\bar{M}=97\%,98\%,99\%{\bar{M}}_{\max}$ at $\bar{t_{0}}=20$.

Therefore, we adopt the weakest chain principle as the crack criterion of the secondary fracture. The key to the criterion is that the secondary fracture will take place at the point of the maximum value of the excited bending moment ${\bar{M}}_{\max}$. However, in our model ${\bar{M}}_{\max}$ is an asymptotic extremum which is actually unreachable through the whole bent process. Therefore, we propose three typical value of $97\%,98\%,99\%{\bar{M}}_{\max}$, the contour curves of which are illustrated in Figure 7, Figure 8 and Figure 9, respectively, when $\bar{t_{0}}=1,10,20$ with variables of distance and time.

Any point in a contour curve reaches the set value with the corresponding spacial coordinate and time coordinate, and the point in the contour curve who owns the minimum value of time is believed to be the case when and where a secondary fracture is about to happen, and is marked. It can be seen from Figure 7 that when $\bar{t_{0}}=1$, the fracture distances are 5.6, 6.3, and 7.8 times nondimensional length or radii of gyration respectively with progressive set critical moment from $97\%{\bar{M}}_{\max}$ to $99\%{\bar{M}}_{\max}$, which indicates that greater set critical value of bending moment leads to greater value of fracture distance, and vice versa, and Figure 8 and Figure 9 share the same fact with greater values of $\bar{t_{0}}$. Meanwhile, the fracture time are 3.1, 3.7, and 5.3 times nondimensional time, respectively, when $\bar{t_{0}}=1$, which indicates that greater set critical value of bending moment as well leads to greater value of fracture time, and vice versa, and cases with greater values of $\bar{t_{0}}$ share the same fact.

It also can be seen from the three contour curves that when the set value is the same, for instance $99\%{\bar{M}}_{\max}$, the fracture distance are 8, 25, and 35 times nondimensional length or radii of gyration and the fracture time are 553,107 times nondimensional time at $\bar{t_{0}}=1,10,20$, respectively, indicating that greater value of $\bar{t_{0}}$ leads to greater value of fracture distance and fracture time, and vice versa.

**Figure 10:**
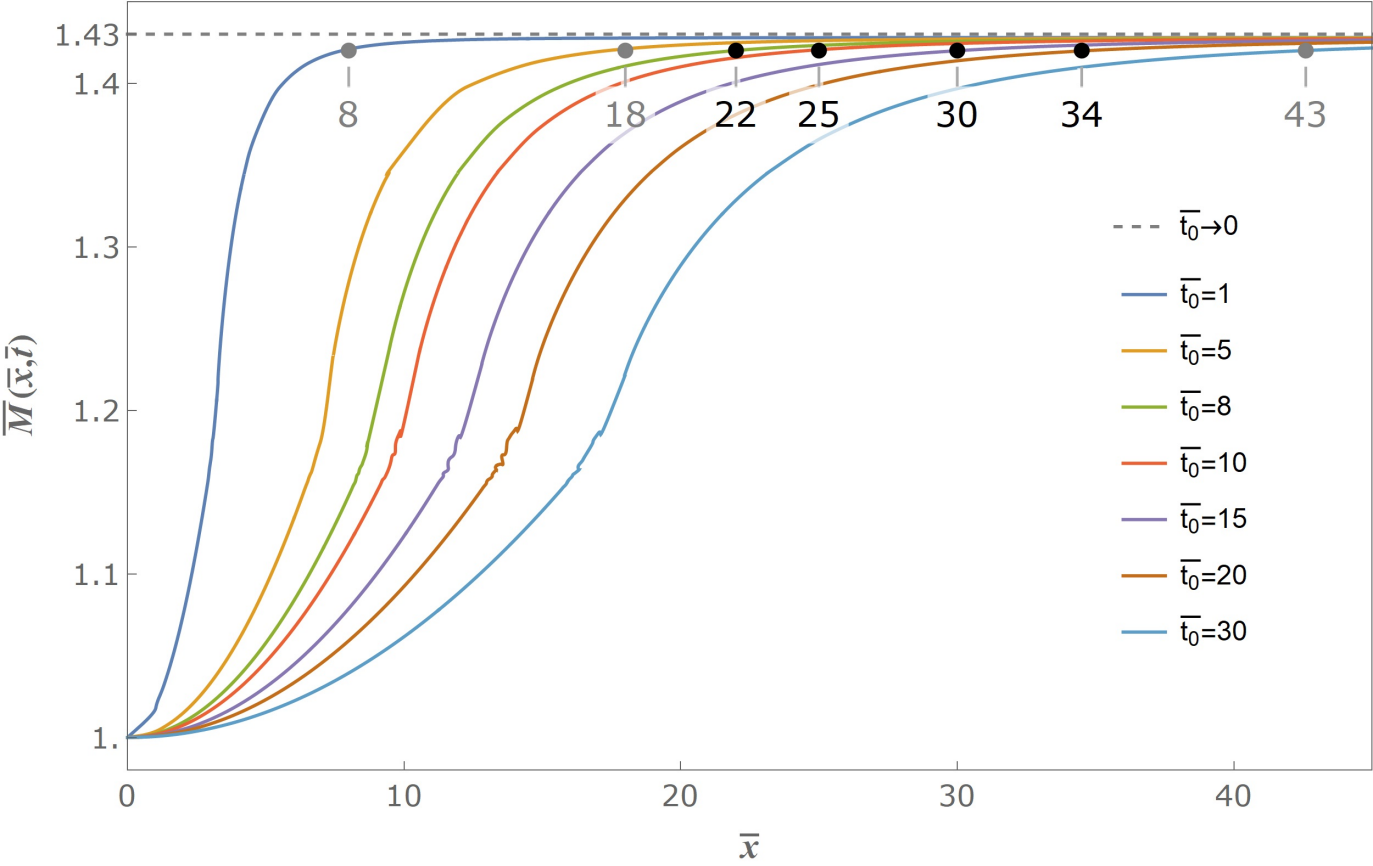
Envelope schematic of maximum excited bending moment with different $\bar{t_{0}}$.

As discussed above, we investigate the effect and influence of release declining time on the secondary fracture process from one perspective of the contour curves with variables of distance and time, and subsequently we are going to continue to investigate from another perspective of the envelope curves of the maximum excited bending moment from the excited flexural waveform curves such as Figure 4, Figure 5 and Figure 6. In fact, we provide Figure 10 of envelope curves extended to seven different values of $\bar{t_{0}}$ along spatial distance. We can see from the curves that whatever value $\bar{t_{0}}$ takes, as long as it is non-zero, the maximum bending moment will always progressively increase and then to the asymptotic extremum value of 1.43 times initial value. However, greater value of $\bar{t_{0}}$, or longer release declining time duration, leads to farther distance when the excited moments reach to some certain set value marked in our schematic, and vice versa.

Meanwhile, Figure 10 reveals improvement and advantage between solutions to our gradual release model and the sudden release model, that by introducing a non-zero valued release declining time $\bar{t_{0}}$, the excited maximum bending moment will not directly increase to its asymptotic extremum, but will undergo a certain time duration and distance before get to some set critical value. Therefore, a characteristic time parameter is brought into solution when the release declining time $\bar{t_{0}}$ is brought to the boundary condition, which is an adjustable time parameter to indicate further understanding towards secondary fracture during spaghetti bent break. As a result, as long as we give a crack criterion of the secondary fracture in terms of bending moment, we are able to get the fracture distance and fracture time from our solution, which are also the characteristic fragmentation length and the characteristic fragmentation time of the secondary fracture.

### 3.2. Estimated Value of $\bar{t_{0}}$

Subsequently, let us discuss what value of $\bar{t_{0}}$ should be taken physically and theoretically.

According to the linear elastic theory, the ultimate crack speed is Rayleigh wave speed ($c_{R}$). However, Schardin and Struth [16] found that the maximum velocity reached by the fast impact crack is material characteristic speed and less than the Rayleigh wave speed. This result was confirmed in other materials using different measurement methods, such as for noncrystalline materials with crack speed range of 0.4 to 0.7$c_{R}$ [17], and for crystal materials ranging from 0.63 to 0.90$c_{R}$ [18]. These studies showed that the ultimate crack speed is constant for each material and occupies a specific proportion in the elastic wave speed.

For linear elastic material of positive Poisson’s ratio, the Rayleigh wave speed equals 0.862–0.955 times of the shear wave speed ($c_{s}$). Moreover, the shear wave speed is about 0.577–0.707 times the elastic wave speed ($c_{0}$). Therefore, the Rayleigh wave speed $c_{R}$ equals to 0.497–0.675 times the elastic wave speed $c_{0}$.

This means that the crack speed is about 0.199–0.473 of the elastic wave speed for noncrystalline material such as spaghetti sticks. As a result, the time duration of the first fracture during spaghetti bent break is the stick thickness *h* divided by crack speed,

(43) $$h/\left(0.199-0.473\right)c_{0}=4R/\left(0.199-0.473\right)c_{0}=\left(8.5-20.1\right)\bar{t}$$

The result in Equation (43) is just the release declining time in our model, or the estimated value of $\bar{t_{0}}$. Therefore, under linear elastic theory, the value of $\bar{t_{0}}$ should be taken as 8–20 times nondimensional time.

Moreover, in fact, the research of the dynamic fracture process of a long rod subjected to pure bending, mainly the first fracture, was originated from Freund and Herrmann’s work [19]. They reported that the crack tip rapidly accelerates to near the characteristic terminal speed, maintains this speed to travel through most of the stick thickness *h*, and then decelerates quickly. They also presented that the bending moment on the fracture section decreases monotonically down to zero with a time duration of $5c_{0}t/h$, which is $20c_{0}t/R$ or 20 times the nondimensional time in our model. Later works by Adeli [20] and Levy [21] further investigated the dynamics of this process and shared the same results of the crack tip transmission time and the moment declining time. In addition, numerical simulation results by the authors suggests the time duration of the first fracture of brittle elastic material subjected to four points bending is 17 times nondimensional time.

As a result, that the value of $\bar{t_{0}}$ is estimated as 8–20 is reasonable, and we can figure out from Figure 10 that the fracture length between the first and second crack is 22–34 times nondimensional length or 6–9 times stick thickness accordingly, when $99\%{\bar{M}}_{\max}$ is chosen as the set critical value.

### 3.3. Experiments

**Figure 11:**
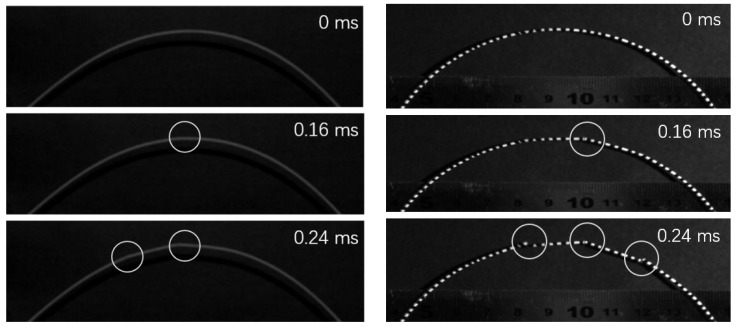
Experimental process of spaghetti break bent.

Experimental bent break processes of spaghetti stick are illustrated in Figure 11. In our experiments, the spaghetti sticks were from the traditional spaghetti pasta of Barilla No.5, with length 240±5mm and diameter 1.72±0.02mm. Image recordings were performed using high-speed camera FASTCAM SA1.1 (Photron, Tokyo, Japan) with resolution of 768×640 and shooting speed of 12,000 fps. Experiments showed that the first fracture usually took place at a point near the middle of the stick, and continued to behave secondary fractures after a certain time and distance from the point of first fracture, suggesting an approximation of 6–13 times stick thickness between the first and the second crack, which is in general accordance with data in Figure 10 when $99\%{\bar{M}}_{\max}$ is chosen as the set value for the secondary fracture, and are also in good agreement with data reported in [15].

Therefore, we adopt $99\%{\bar{M}}_{\max}$ as the set value of the secondary fracture during spaghetti bent break. Subsequently, we can figure out the fragmentation length between the first and the second crack is 22–34 times nondimensional length or 6–9 times stick thickness from Figure 10 when we adopt 8–20 as the estimated value of release declining time.

## 4. Conclusions

In this paper, we attempted to further understand the secondary fracture during spaghetti bent break.

We propose a half-infinite model that mimics the physics, introduces a gradual release boundary condition—a linearly release declining time $t_{0}$, and thus brings an adjustable time parameter related to fragmentation length and fragmentation time to our model, which improves previous sudden release model, fixes the defects, and agrees better with the real situation.

We derive a specific analytical solution in terms of excited bending moment using Euler–Bernoulli beam theory, and point out that the gradually declined release of the initial moment leads to a burst of flexural stress waves, and these waves locally increase the excited bending moment in the stick and progressively to get to the maximum value, and eventually leads to the secondary fracture.

The excited moments increase with time and distance, and have an asymptotic extremum value of 1.43 times initial moment. Unlike the sudden release case, the gradual release in our model requires and gives certain distance and time when the excited bending moment reaches its extremum value, which results in a possibility to predict the detailed fracture parameters such as fragmentation length and time of the secondary fracture.

We suggest 8–20 as reasonable values of gradual declining release time $\bar{t_{0}}$ and $99\%{\bar{M}}_{\max}$ as the critical value of the secondary fracture, and as a result, the fragmentation length between the first and secondary fracture is 6–9 times greater than the stick thickness, which is in general accordance with experimental data.

## Data Availability

Data sharing not applicable.

## References

[1] Miklowitz J. (1953). Elastic waves created during tensile fracture—The phenomenon of a second fracture. J. Appl. Mech..

[2] Phillips J.W. (1970). Stress pulses produced during the fracture of brittle tensile specimens. Int. J. Solids Struct..

[3] Kolsky H. (1973). The stress pulses propagated as a result of the rapid growth of brittle fracture. Eng. Fract. Mech..

[4] Kinra V. (1976). Stress pulses emitted during fracture in tension. Int. J. Solids Struct..

[5] Bodner S.R. (1973). Stress waves due to fracture of glass in bending. J. Mech. Phys. Solids.

[6] Kinra V., Kolsky H. (1977). The interaction between bending fractures and the emitted stress waves. Eng. Fract. Mech..

[7] Schindler H.J., Kolsky H. (1983). Multiple fractures produced by the bending of brittle beams. J. Mech. Phys. Solids.

[8] Sykes C. (1996). No Ordinary Genius.

[9] Nickalls O., Nickalls R. (1995). Linear Spaghetti. New Sci..

[10] Nickalls O., Nickalls R. (1998). Pasta Puzzle. New Sci..

[11] Audoly B., Neukirch S. (2005). Fragmentation of rods by cascading cracks: Why spaghetti does not break in half. Phys. Rev. Lett..

[12] Corrales T.P., Friedemann K., Fuchs R., Roy C., Crespy D., Kappl M. (2016). Breaking nano-spaghetti: Bending and fracture tests of nanofibers. Langmuir.

[13] Graff K.F. (1975). Wave Motion in Elastic Solids.

[14] Oberhettinger F., Badii L. (1973). Tables of Laplace Transforms.

[15] Heisser R.H., Patil V.P., Stoop N., Villermaux E., Dunkel J. (2018). Controlling fracture cascades through twisting and quenching. Proc. Natl. Acad. Sci. USA.

[16] Schardin H., Struth W. (1938). Hochfrequenzkinematographische untersuchung der bruchvorgänge in glas. Glastech. Berichte.

[17] Paxson T.L., Lucas R.A., Broberg B., Kobayashi A., Rosenfield A.R. (1973). An experimental investigation of the velocity characteristics of a fixed boundary fracture model. Proceedings of an International Conference on Dynamic Crack Propagation.

[18] Washabaugh P.D., Knauss W.G. (1993). Non-steady, periodic behavior in the dynamic fracture of PMMA. Int. J. Fract..

[19] Freund L.B., Herrmann G. (1976). Dynamic Fracture of a Beam or Plate in Plane Bending. ASME J. Appl. Mech..

[20] Adeli H., Herrmann G., Freund L.B. (1977). Effect of Axial Force on Dynamic Fracture of a Beam or Plate in Pure Bending. ASME J. Appl. Mech..

[21] Levy C., Herrmann G. (1982). Effect of Shear and Rotary Inertia on Dynamic Fracture of a Beam or Plate in Pure Bending. ASME J. Appl. Mech..

